# The Case to Case Comparison of Hormone Receptors and HER2 Status between Primary Breast Cancer and Synchronous Axillary Lymph Node Metastasis

**DOI:** 10.31557/APJCP.2020.21.6.1559

**Published:** 2020-06

**Authors:** Sasithorn Sujarittanakarn, Wanwisa Himakhun, Worawarn Worasawate, Wilairat Prasert

**Affiliations:** 1 *Department of Surgery, Faculty of Medicine, Thammasat University, Pathumthani, Thailand. *; 2 *Department of Pathology and Forensic science, Faculty of Medicine, Thammasat University, Pathumthani, Thailand. *; 3 *Department of Surgery, Thammasat Hospital, Pathumthani, Thailand. *

**Keywords:** ER- PR- HER2, primary breast cancer, synchronous axillary lymph node

## Abstract

**Background:**

Nowadays, the adjuvant treatment for breast cancer patients chosen depends on immunohistochemical pattern of Estrogen receptor(ER), Progesterone receptor(PR) and HER2 status of primary breast tumor. Several retrospective studies showed significant discordance in receptor expression between primary and metastatic tumors. The objective of this research was to determine discordant rate of ER, PR and HER2 status between primary breast cancer and synchronous axillary lymph node metastasis of individual breast cancer patients in Thammasat University Hospital.

**Methods:**

A prospective observational study of all breast cancer patients who have axillary metastasis and underwent surgery at Thammasat Hospital between January 2011 to December 2015. Tumor staging, ER, PR, and HER2 status on primary breast tumor were recorded. Synchronous axillary lymph node metastasis was evaluated with immunohistochemistry for ER, PR, and HER2.

**Results:**

The ER-positive rate from primary tumor to synchronous axillary lymph node metastasis decreased from 74.7% to 71.7%; the HER2 overexpression rate was decreased from 26% to 24%. In contrast, PR positive rate were 71% in both primary tumor and synchronous axillary lymph node metastasis. In case to case comparison, discordance rate of ER, PR and HER2 status between primary breast cancer and synchronous axillary lymph node metastasis were 11.1%, 20.2% and 10.1%, respectively. Furthermore, the tumor staging was not significant associated with discordance of ER, PR and HER2.

**Conclusion:**

ER, PR and HER 2 biomarkers showed significant concordance between primary tumor and synchronous axillary lymph node metastasis. Hence, if we cannot assess the ER, PR and HER2 status in primary tumor, then synchronous axillary lymph node metastasis can be studied instead. However, the repeat of biomarker testing in node-positive breast cancer patients may be beneficial for tailored adjuvant therapy, especially for patients with negative hormone receptor and/or HER2 profile on primary tumor.

## Introduction

Breast cancer is the most common cancer in women in worldwide included Thailand. The determination of estrogen receptor (ER), progesterone receptor (PR), human epidermal growth factor receptor (HER2), and Ki-67 were useful for defining subtypes of breast cancer which provided prognostic information and generally sufficient to guide adjuvant systemic treatment for patients (Curigliano et al., 2017). The American Society of Clinical Oncology and College of American Pathologist (ASCO/CAP) developed widely adopted evidence-based guidelines that were published in 2010. They recommended ER and PR be measured on all invasive breast cancers. ER and PR status are determined by immunohistochemistry (IHC), while HER2 status is determined by IHC and/or in situ hybridization assays (Hammond et al., 2010; Wolff et al., 2014). Most of the clinical researches, a molecular profile was evaluated on the primary tumor. Synchronous axillary lymph node metastasis is one of the important metastatic routes of breast carcinoma (Frederick, 2014) and may represent the potentially recurrent or metastatic breast cancer cells much better than the primary carcinomas. However, several studies had demonstrated ER, PR and HER2 status instability between primary breast cancer and synchronous axillary lymph node metastasis (Aitken et al., 2010; Rossi et al., 2015). Furthermore, some article recommended to determination of hormone receptors and HER2 status in both primary tumor and synchronous axillary nodal metastasis to guide therapy management and evaluate the recurrent risk also (Lower et al., 2017; Georgescu et al., 2018). 

Our study we aimed to investigate the percentage of discordance of ER, PR and HER2 between primary breast cancer and synchronous axillary lymph node metastasis in Thammasat University Hospital. Moreover, we aimed to assess the relation of discordance of ER, PR and HER2 on staging of breast cancer. 

## Materials and Methods

*Patients*

Breast carcinoma patients with synchronous axillary lymph node metastases who underwent breast and axillary surgeries at Thammasat University hospital, Pathumthani, Thailand, were selected for study between January 2011 and December 2015. Exclusion criteria were recurrent breast cancer, post-neoadjuvant treatment specimens and poorly preserved specimens. Patients’ information was collected on SPSS. All the patients were recorded as alive at their last known follow-up date. 

*Histopathology Evaluation*

Immunohistochemistry for ER, PR and HER-2 were performed on paraffin blocks of primary tumor and synchronous axillary lymph node metastasis. The tissue sections were sliced to 5-6 microns then mounted on glass slides. For ER, PR and HER-2 staining, all procedures were performed using an Ventana Bench Mark-XT automated slide stainer(Ventana, Tuscon, USA) procedure: XT ultraView DABv3, with anti-ER(SP1) rabbit monoclonal antibody (Ventana), anti-PR(clone1E2) rabbit monoclonal antibody (Ventana) and anti-HER2/neu (clone4B5) rabbit monoclonal antibody (Ventana). The scoring system was performed upon The American Society of clinical oncology(ASCO) and The College of American Pathologist(CAP) advises by one breast pathologist of Thammasat University Hospital. 

**Figure 1:**
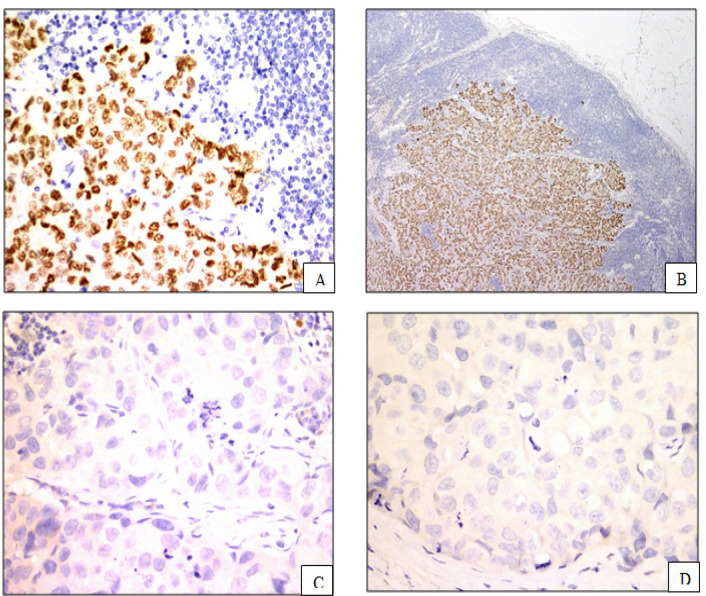
Immunohistochemical Analysis of Estrogen Receptor (ER) and Progesterone Receptor (PR); A, ER positive; B, PR positive; C, ER negative; D, PR negative

For ER and PR interpretation, the “Positive” result were interpreted when percentage of invasive tumor nuclei were at least 1%. If the tumor nuclei staining are less than 1%, the result was “Negative” (Hammond et al., 2010) (Figure 1). 

**Figure 2:**
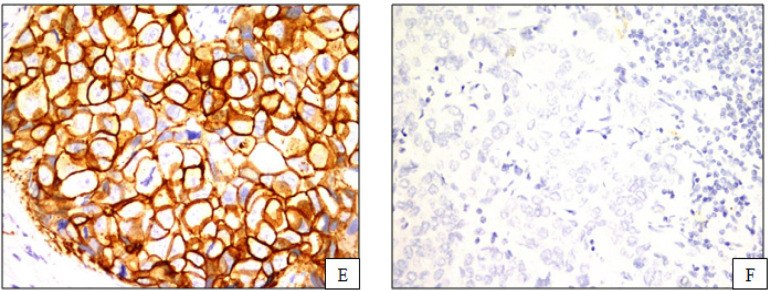
Immunohistochemical Analysis of Human Epidermal Growth Factor Receptor (HER2); E, HER2 positive; F, HER2 negative

For HER2 interpretation, the “Positive” IHC3+ were circumferential membrane staining that is complete, intense and within >10% of tumor cells. The “Equivocal” IHC2+ were circumferential membrane staining that is incomplete and/or weak/moderate and within >10% of tumor cells or complete and circumferential membrane staining that is intense and within ≤10% of tumor cells. The “Negative” IHC1+ were incomplete membrane staining that is faint within >10% of tumor cells. The “Negative” IHC 0 were no staining observed or incomplete and faint membrane staining within ≤10% (Wolff et al., 2014). (Figure 2) 

*Data collection*

Information was collected on a SPSS. Patient’s age, gender, size of primary tumor, histology of primary tumor, grade of tumor, number of involved lymph nodes, status of distant metastases, expression of ER, PR and HER2 on primary and metastatic tumor in axillary lymph nodes, recurrent status and survival status were recorded. 

*Statistical Analysis*

The Mcnemar exact probability test was used to evaluate whether the differences in dichotomized variables measured in the present study in both direction (+/- and -/+) were equally common when comparing primary tumors and synchronous axillary lymph node metastases. The Logistic regression analysis was used to evaluate whether the tumor differentiation and staging as risk factors of discordance breast carcinoma patients. P-values less than 0.05 was considered significant. The statistical software package Stata 15.1 (StataCorp., IBM, SPSS, USA) was used for statistical calculations. 

## Results

A total of 120 breast carcinoma patients with synchronous axillary lymph node metastases who underwent breast and axillary surgeries in Thammasat University Hospital between January 2011 and December 2015 were enrolled in this study. We excluded 4 post neoadjuvant treatment patients and 17 patients whose specimens were unavailable. Immunohistochemical stains were performed on 99 cases. The patient’s age ranged between 21 and 85 years (average 55.23 years). Most of the patients (94%) had invasive ductal carcinomas, 2% had invasive lobular carcinomas, 2% had mixed ductal and lobular carcinomas, 1% had neuroendocrine tumor and 1% had mucinous carcinoma. In terms of tumor differentiation, 50.5% of patients had poorly differentiation tumors, 41.4% had moderate differentiation and 8.1% had well differentiation (Table 1). 

**Figure 3:**
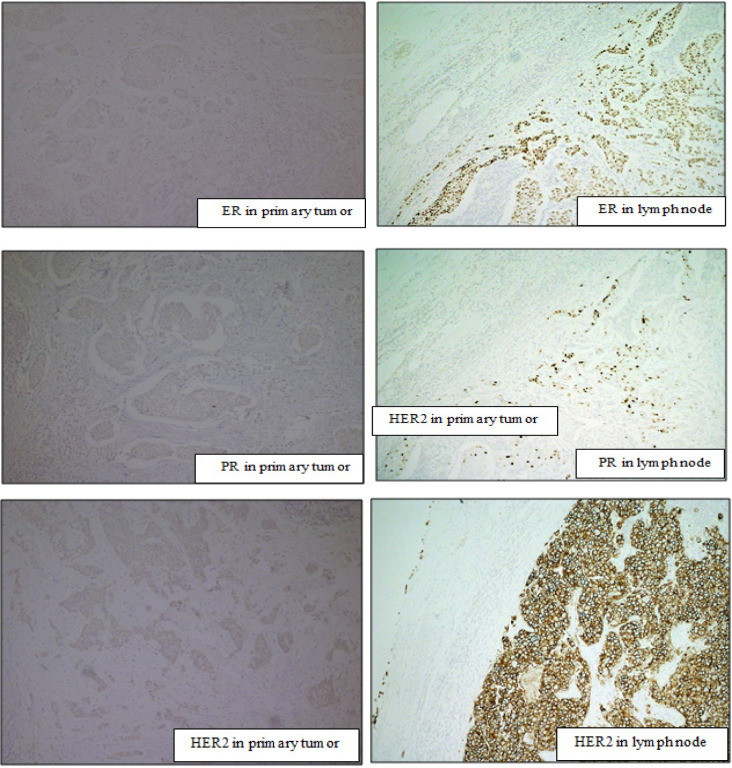
This Patient is 46 Years Old Women with pT1N1M0 Invasive Ductal Carcinoma, Nuclear Grade 3. In primary tumor showed triple-negative (ER 0%, PR 0%, and HER2 score1+) but positive ER, PR, and HER2 in metastatic lymph node (ER 80%, PR 5%, and HER2 score 3+).

The hormonal receptor status was compared between primary tumor and metastatic tumor in synchronous axillary lymph node. Estrogen receptor positivity was observed in 74.7% of primary breast tumors in comparison with estrogen receptor positivity in metastatic synchronous axillary lymph node which was 71.7% (P value = 0.549). Progesterone receptors were positive in 71.7% of primary tumors which was same as in that of metastatic axillary lymph nodes (P value = 1.000). The HER2 receptor results in our study demonstrated 20 cases with equivocal result (intensity stain of 2+). Thus, the total number used to calculate the HER2 result in our study was instead 79 cases. HER2 expression in primary tumors and metastatic axillary lymph nodes was positive in 26.3% and 24.2% respectively (P value = 0.754). These P values confirm that there was no statistically significant difference in biomarker expression between primary breast tumor and metastatic tumor in synchronous axillary lymph nodes (Table 2). Figure 3 showed the patient in our study who presented triple-negative in the primary tumor while all positive in a metastatic lymph node.

Based on a case to case comparison, 67.6% of the cases had positive estrogen receptors in both primary tumor and lymph nodes, while 21.2% of the cases had negative estrogen receptors in both primary tumor and lymph nodes. Therefore 88.9% of the cases had concordance results in estrogen receptor expression. Progesterone receptors showed 61.6% positivity in both primary and lymph node samples while 18.2% of the cases were negative in both primary tumor and lymph node, resulting in a 79.8% agreement in progesterone receptors expression (Table 3). According to the ASCO/CAP guideline, the same equivocal HER2 immunohistochemistry specimen should further be investigated with in situ hybridization(ISH). Unfortunately, we cannot afford the cost for ISH in our study due to budget limitation. In our study, 20 patients demonstrated equivocal HER2 results, thus, the remaining total number of patients without equivocal results was 79 patients. *HER2* expression was positive in both the primary tumor and lymph node in 24% of the cases and negative in 65.8% of the cases, making the total concordance result 89.9%. Meanwhile, if equivocal patients were included with the positive results, the discordance rate of HER2 will be 21% (p=0.0011), but if the equivocal results were added to the negative results, the discordance rate of HER2 would instead be 12% (p=0.5637). In other words, the discordance rates of estrogen receptors, progesterone receptors and HER2 in our study were 11.1%, 20.2% and 10.1%, respectively.

For relation of discordance of ER, PR and HER2 with staging of breast cancer. In the ER discordance group, 5 patients had stage 2 breast cancer and 6 patients had stage 3 or 4 breast cancer. Whereas the PR discordance group included10 patients with stage 2 breast cancer and 10 patients with stage 3 or 4 breast cancer. The HER2 discordance group had either stage 2 and 3 or 4 breast cancer had 4 patients. A comparison of staging showed no significant difference between discordance and concordance groups (Table 4). 

**Table 1:** Primary Tumor Characteristics

Total No. of patient	N = 99
Tumor size	
Range	1.0 to 8.5 cm
Mean	3.32 ± 1.60 cm
Tumor type	
Invasive ductal carcinoma	93 (94%)
Invasive lobular carcinoma	2 (2%)
Other	4(4%)
Tumor grading	
Well differentiation	9 (9.1%)
Moderate differentiation	41 (41.4%)
Poorly differentiation	49 (49.5%)

**Table 2:** Comparison of Immunohistochemistry between Primary Breast Tumor and Metastatic Lymph Nodes

Biomarkers	Primary Breast Tumor No (%)	Lymph node metastasis No (%)	*P-*value
Total no of patient	99	99	
Estrogen receptor(ER)			
Positive	74 (74.7%)	71 (71.7%)	0.549
Negative	25 (25.3%)	28 (28.3%)	
Progesterone receptor(PR)			
Positive	71 (71.7%)	71 (71.7%)	1
Negative	28 (28.3%)	28 (28.3%)	
HER2 status			
Positive	26 (26.3%)	24 (24.2%)	0.754
Negative	55 (55.5%)	70 (70.7%)	
Equivocal	18 (18.2%)	5 (5.1%)	

**Table 3:** Case by Case Comparison of Immunohistochemistry between Primary Breast Tumor and Metastatic Lymph Node

Biomarkers	Estrogen receptor(ER)	Progesterone receptor(PR)	HER2 status
Positive in both primary tumor and lymph node	67	61	19
Negative in both primary tumor and lymph node	21	18	52
Total concordance	88 (88.9%)	79 (79.8%)	71 (89.9%)
Positive in primary tumor but negative in lymph node	7	10	5
Negative in primary tumor but positive in lymph node	4	10	3
Total discordance	11 (11.1%)	20 (20.2%)	8 (10.1%)
Total	99	99	79
*p*-value McNemar’s chi-square	0.3657	1	0.4795

**Table 4:** Relation of Discordance of ER, PR and HER2 with Breast Cancer Staging

Markers		Stage 2 N(%)	Stage 3/4 N(%)	*P*-value
ER	Discordance	5 (11)	6 (11)	1.000
	Concordance	39 (89)	49 (89)	
PR	Discordance	10 (23)	10 (18)	0.576
	Concordance	34 (77)	45 (82)	
HER2 [2+ omitted]	Discordance	4 (12)	4 (9)	0.708
	Concordance	28 (88)	43 (91)	

## Discussion

Breast cancer cells may disseminate by either the lymphatic or the vascular system. Approximately 97% of the lymph from the breast flows to the axillary nodes, whereas the remaining 3% flows to the internal mammary chain (Hultborn and Raghnult, 1995). In other words, axillary lymph nodes are the most important metastasis routes of breast cancer. After surgical treatment, most breast cancer patients need adjuvant systemic treatment to reduce the risk of local/systemic recurrence and prolong survival (Frederick, 2014). Adjuvant systemic treatment for breast cancer consists of chemotherapy, endocrine therapy, and targeted therapy. In order to determine the optimal type and sequence of systemic treatment, several variables are considered including the disease stage, functional status of patients, clinicopathologic and the molecular subtype of the tumor. Special consideration is given to the expression of hormone receptor and HER2 status, as they are universal indicators of efficacy and play a role in determining the prognosis of breast cancer. Hormonal status is one of the standard predictive factors in determining whether endocrine therapy is indicated (Hammond et al., 2010). The NCCN guideline suggests patients with invasive breast cancer that are ER or PR positive should be considered for adjuvant endocrine therapy. The lack of benefit from endocrine therapy for women with ER-negative invasive breast cancer has been confirmed in large overviews of randomized clinical trials. 

Furthermore, the St. Gallen International Expert Consensus Conference has for years led to the development of tailored treatment based on clinical and biological subsets of breast cancer. In broad clinical terms, there are four subtypes of breast cancer that solicit distinct treatment approaches: triple-negative tumors, HER2 positive tumors and two types of ER-positive breast cancers (Curigliano et al., 2017). In both routine clinical practice and research studies, the management of patients is frequently based on the biomarker characteristics of the primary tumor. In addition, the NCCN Panel recommends that metastatic disease at presentation or first recurrence of disease should be biopsied so that the ER, PR and HER2 status is determined as a part of the workup for patients with recurrent or stage IV disease (Arslan et al., 2011; Gradishar et al., 2018). This is because hormonal receptors and HER2 status may change with time as the disease progresses from primary tumor to metastatic lesions (Maynadier et al., 2008; Huang et al., 2009). This heterogeneity might be the cause of treatment failure since the distant disease is more likely to be the target for adjuvant systemic therapy after local treatment such as surgery and radiotherapy (Aitken et al., 2010). The reasons for the discordance may relate to changes in biology of the disease, differential effect of prior treatment on clonal subsets, tumor heterogeneity, or imperfect accuracy and reproducibility of assays (Pusztai et al., 2010). The discordance rates range between 3.4% to 60% for ER-negative to ER-positive; 7.2% to 31% for ER -positive to ER-negative and 0.7% to 11% for HER2 (Gong et al., 2005; Bogina et al., 2011). 

The synchronous axillary lymph node metastases potentially provide a better representation of the metastatic cell population in comparison to the primary tumor. Several studies have been done comparing the ER, PR and HER2 status of primary tumors and paired metastasis. Aitken et al., (2010) studied 211 patients with invasive primary breast carcinomas along with paired axillary lymph nodes using quantitative analysis to assess changes in ER, PR, and HER2 receptors. Overall, 46.9% of the cases had incongruent breast/node receptor status of at least one receptor. The discordance rate of ER, PR, and HER2 were 28.3%, 23.4%, and 8.9%, respectively. In addition, of the 55 ER discordant patients, 20 cases (10.3%) switched from ER-negative to ER-positive and 35 cases (18%) from ER-positive to ER-negative. The *PR* expression also followed a similar trend, as out of 45 patients (23.4%) with PR discordant, 17 cases (8.8%) switched from PR negative to PR positive and 28 cases (14.6%) from PR positive to PR negative. A higher proportion of HER2 retained their original status. A significant number of patients demonstrated discordant quantitative molecular marker expressions primary and nodal disease, which may be an alternative explanation for therapeutic resistance to targeted therapy in breast cancer. Another study conducted by Nedergaard et al., (1995) investigated the ER status in 101 primary breast cancers and their axillary lymph node metastases. The concordance rate of the ER status was 79% and the discordance rate was 21%. The discordant ER status, may be due to the loss of ERs in the metastatic cells or tumor heterogeneity, which in turn, could explain the cause of failure of endocrine therapy in some patients involving ER-positive primary tumors. In contrast, Muhammad et al., determined the ER, PR and HER2 status on primary breast cancers and axillary lymph node metastasis in 100 patients (Azam et al., 2009). Based on a case to case comparison of ER, PR and HER2 receptors, significant concordance rates were demonstrated between primary and metastatic breast carcinoma at axillary lymph nodes as 91%, 88%, and 95%, respectively.

Based on a case to case comparison, the discordance rates obtained from this study for ER, PR and HER2 receptors were 11.1%, 20.2%, and 10.1%, respectively. Out of 11 patients with ER discordance, 7 patients showed ER positive results in primary tumors but negative results in lymph nodes, whereas 4 patients had ER negative in primary tumors but were instead positive in lymph nodes. Meanwhile, out of 20 patients from the PR discordant group, 10 patients were PR positive in primary tumors but negative in lymph nodes and another 10 patients had PR-negativity in primary tumors but were positive in lymph nodes. This may partly alter management and improve the patient’s prognosis to potentially prevent recurrence or metastasis. Patients with receptor alteration from hormone receptor-negative primary tumors to hormone receptor-positive synchronous axillary metastasis, may benefit from receiving endocrine therapy and potentially have a better prognosis. The change in hormone receptor profile in axillary metastasis may explain why some patients with ER-negative and/or PR-negative primary tumors have been reported to respond to endocrine therapy. On the other hand, this may explain why some hormone receptor-positive tumors did not respond to endocrine therapy. Out of 8 patients from the HER2 discordant group, 5 patients had HER2 positive primary tumors but negative lymph nodes, while 3 patients had HER2 negative primary tumors and positive lymph nodes. HER2-targeted therapy in patients with receptor alteration from HER2-positive primary tumors to HER2-negative axillary metastasis, may cause more harm than benefit, as well as being ineffective and costly. On the contrary, patients who change from HER2-negative primary tumors to HER2-positive axillary metastasis may benefit from the administration of HER2-targeted therapy. Having assessed the discordance rate of ER, PR, and HER2 status between primary breast cancer and synchronous axillary lymph node metastasis, our study demonstrated no significant difference between discordance and concordance groups. 

However, the effectiveness of treatment selection according to the biomarker of synchronous axillary lymph nodes cannot yet be proven. Further studies will be needed to determine the potential role of routine repeat testing of ER, PR and HER2 status and other potential factors, which may cause discordance results and therefore require repeat biomarker testing.

In conclusion, the discordance of ER, PR and HER2 between primary tumors and synchronous axillary lymph node metastasis occurred in 10.1%-20.2% of our cases. The repeat of biomarker testing in node-positive breast cancer patients may be beneficial, especially for patients with negative hormone receptor and/or HER2 profile but positive results in synchronous axillary lymph nodes as patients could become eligible for hormonal treatment and/or HER2-targeted therapy. Hence, may significantly improve the patient’s outcome. Based this study, results obtained demonstrate significant concordance, thus implying that synchronous axillary lymph nodes can be used for biomarker testing if the primary tumor is not available to testing as an alternative source. 
